# A Public Health Research Agenda for Managing Infodemics: Methods and Results of the First WHO Infodemiology Conference

**DOI:** 10.2196/30979

**Published:** 2021-09-15

**Authors:** Neville Calleja, AbdelHalim AbdAllah, Neetu Abad, Naglaa Ahmed, Dolores Albarracin, Elena Altieri, Julienne N Anoko, Ruben Arcos, Arina Anis Azlan, Judit Bayer, Anja Bechmann, Supriya Bezbaruah, Sylvie C Briand, Ian Brooks, Lucie M Bucci, Stefano Burzo, Christine Czerniak, Manlio De Domenico, Adam G Dunn, Ullrich K H Ecker, Laura Espinosa, Camille Francois, Kacper Gradon, Anatoliy Gruzd, Beste Sultan Gülgün, Rustam Haydarov, Cherstyn Hurley, Santi Indra Astuti, Atsuyoshi Ishizumi, Neil Johnson, Dylan Johnson Restrepo, Masato Kajimoto, Aybüke Koyuncu, Shibani Kulkarni, Jaya Lamichhane, Rosamund Lewis, Avichal Mahajan, Ahmed Mandil, Erin McAweeney, Melanie Messer, Wesley Moy, Patricia Ndumbi Ngamala, Tim Nguyen, Mark Nunn, Saad B Omer, Claudia Pagliari, Palak Patel, Lynette Phuong, Dimitri Prybylski, Arash Rashidian, Emily Rempel, Sara Rubinelli, PierLuigi Sacco, Anton Schneider, Kai Shu, Melanie Smith, Harry Sufehmi, Viroj Tangcharoensathien, Robert Terry, Naveen Thacker, Tom Trewinnard, Shannon Turner, Heidi Tworek, Saad Uakkas, Emily Vraga, Claire Wardle, Herman Wasserman, Elisabeth Wilhelm, Andrea Würz, Brian Yau, Lei Zhou, Tina D Purnat

**Affiliations:** 1 Directorate for Health Information & Research Ministry for Health Valetta Malta; 2 WHO Regional Office for Africa Brazzaville Congo; 3 US Centers for Disease Control and Prevention Atlanta, GA United States; 4 WHO Regional Office for Eastern Mediterranean Cairo Egypt; 5 Department of Psychology College of Liberal Arts & Sciences University of Illinois Urbana-Champaign Champaign, IL United States; 6 Department of Communications World Health Organization Geneva Switzerland; 7 WHO Regional Office for Africa Dakar Senegal; 8 Department of Communication Sciences and Sociology Communication Sciences Faculty University Rey Juan Carlos Madrid Spain; 9 Faculty of Social Sciences and Humanities Universiti Kebangsaan Malaysia Bangi Malaysia; 10 Department of Communication Budapest Economics University (BGE) Budapest Hungary; 11 Institute for Information, Telecommunications and Media Law University of Münster (WWU) Münster Germany; 12 DATALAB - Center for Digital Social Research School of Communication and Culture Aarhus University Aarhus Denmark; 13 WHO Regional Office for South East Asia New Delhi India; 14 Department of Infectious Hazards Management Emergency Preparedness Division World Health Organization Geneva Switzerland; 15 Center for Health Informatics School of Information Sciences University of Illinois at Urbana-Champaign Champaign, IL United States; 16 Immunize Canada Canadian Public Health Association Ottawa, ON Canada; 17 Department of Political Science University of British Columbia Vancouver, BC Canada; 18 CoMuNe Lab Fondazione Bruno Kessler Povo Italy; 19 Biomedical Informatics and Digital Health School of Medical Sciences The University of Sydney Sydney Australia; 20 School of Psychological Science The University of Western Australia Perth Australia; 21 European Centre for Disease Prevention and Control Stockholm Sweden; 22 Graphika New York, NY United States; 23 Department of Security and Crime Science University College London London United Kingdom; 24 Ted Rogers School of Management Ryerson University Toronto, ON Canada; 25 Ministry of Health Ankara Turkey; 26 UNICEF Headquarters New York, NY United States; 27 Immunisation and Countermeasures Department Public Health England London United Kingdom; 28 The Faculty of Communication Science Bandung Islamic University (UNISBA) Bandung Indonesia; 29 Oak Ridge Institute for Science and Education Oak Ridge, TN United States; 30 Department of Physics George Washington University Washington, DC United States; 31 Journalism and Media Studies Centre The University of Hong Kong Hong Kong China; 32 Emergency Preaparedness Division World Health Organization Geneva Switzerland; 33 Faculty I Department of Nursing Science II Trier University Trier Germany; 34 Advanced Academic Programs Johns Hopkins University Washington, DC United States; 35 Department of Digital Health and Innovation Science Division World Health Organization Geneva Switzerland; 36 Yale Institute for Global Health Yale University New Haven, CT United States; 37 Usher Institute Edinburgh Medical School University of Edinburgh Edinburgh United Kingdom; 38 British Columbia Centre for Disease Control Vancouver, BC Canada; 39 Department of Health Sciences and Medicine University of Lucerne Lucerne Switzerland; 40 Swiss Paraplegic Research Lucerne Switzerland; 41 Department of Humanities Studies Free University of Languages and Communication IULM Milan Italy; 42 metaLAB (at) Harvard Harvard University Cambridge, MA United States; 43 Office of Infectious Disease Global Health Bureau United States Agency for International Development (USAID) Washington, DC United States; 44 Computer Science Department Illinois Institute of Technology Chicago, IL United States; 45 Masyarakat Anti Fitnah Indonesia (MAFINDO) Jakarta Indonesia; 46 International Health Policy Programme Ministry of Public Health Bangkok Thailand; 47 Science Division World Health Organization Geneva Switzerland; 48 Deep Children Hospital and Research Centre Gandhidham India; 49 Fathm London United Kingdom; 50 Public Health Association of British Columbia Victoria, BC Canada; 51 Vaccine Safety Net (VSN) Geneva Switzerland; 52 Department of History University of British Columbia Vancouver, BC Canada; 53 Faculty of Medicine Mohamed V University in Rabat Rabat Morocco; 54 Hubbard School of Journalism and Mass Communication University of Minnesota Minneapolis, MN United States; 55 First Draft News New York, NY United States; 56 Centre for Film and Media Studies University of Cape Town Cape Town South Africa; 57 Department of Regulation and Prequalification Access to Medicines and Health Products Division World Health Organization Geneva Switzerland; 58 Public Health Emergency Center Chinese Center for Disease Control and Prevention Beijing China

**Keywords:** infodemic, infodemiology, infodemic management, research agenda, research policy, COVID-19, SARS-CoV-2, community resilience, knowledge translation, message amplification, misinformation, disinformation, information-seeking behavior, access to information, information literacy, communications media, internet, risk communication, evidence synthesis, attitudes, beliefs

## Abstract

**Background:**

An infodemic is an overflow of information of varying quality that surges across digital and physical environments during an acute public health event. It leads to confusion, risk-taking, and behaviors that can harm health and lead to erosion of trust in health authorities and public health responses. Owing to the global scale and high stakes of the health emergency, responding to the infodemic related to the pandemic is particularly urgent. Building on diverse research disciplines and expanding the discipline of infodemiology, more evidence-based interventions are needed to design infodemic management interventions and tools and implement them by health emergency responders.

**Objective:**

The World Health Organization organized the first global infodemiology conference, entirely online, during June and July 2020, with a follow-up process from August to October 2020, to review current multidisciplinary evidence, interventions, and practices that can be applied to the COVID-19 infodemic response. This resulted in the creation of a public health research agenda for managing infodemics.

**Methods:**

As part of the conference, a structured expert judgment synthesis method was used to formulate a public health research agenda. A total of 110 participants represented diverse scientific disciplines from over 35 countries and global public health implementing partners. The conference used a laddered discussion sprint methodology by rotating participant teams, and a managed follow-up process was used to assemble a research agenda based on the discussion and structured expert feedback. This resulted in a five-workstream frame of the research agenda for infodemic management and 166 suggested research questions. The participants then ranked the questions for feasibility and expected public health impact. The expert consensus was summarized in a public health research agenda that included a list of priority research questions.

**Results:**

The public health research agenda for infodemic management has five workstreams: (1) measuring and continuously monitoring the impact of infodemics during health emergencies; (2) detecting signals and understanding the spread and risk of infodemics; (3) responding and deploying interventions that mitigate and protect against infodemics and their harmful effects; (4) evaluating infodemic interventions and strengthening the resilience of individuals and communities to infodemics; and (5) promoting the development, adaptation, and application of interventions and toolkits for infodemic management. Each workstream identifies research questions and highlights 49 high priority research questions.

**Conclusions:**

Public health authorities need to develop, validate, implement, and adapt tools and interventions for managing infodemics in acute public health events in ways that are appropriate for their countries and contexts. Infodemiology provides a scientific foundation to make this possible. This research agenda proposes a structured framework for targeted investment for the scientific community, policy makers, implementing organizations, and other stakeholders to consider.

## Introduction

A pneumonia of unknown cause detected in Wuhan, China, was first reported to the World Health Organization (WHO) Country Office in China on December 31, 2019. The disease, caused by a novel coronavirus (SARS-CoV-2), was subsequently named COVID-19, and it was declared a Public Health Emergency of International Concern on January 30, 2020. On March 11, 2020, the WHO characterized the outbreak as a pandemic. Globally, as of August 23, 2021, 211,373,303 confirmed cases of COVID-19, including 4,424,341 deaths, had been reported to the WHO [1].

On February 15, 2020, WHO Director-General Tedros Adhanom Ghebreyesus raised the concern that the epidemic was accompanied by an infodemic [2]. An infodemic is an overflow of information of varying quality that surges across digital and physical environments during an acute public health event and makes it difficult for people to find information to better protect themselves and their communities [3]. An infodemic can lead to confusion, misunderstanding of health information, risk-taking, and behaviors that can harm health, hinder the public health response, and lead to mistrust in health authorities. [4]. Therefore, people need timely, accurate, and accessible information in the right format and amount during epidemics to adopt health-promoting behavior to protect themselves, their families, and their communities against the infection.

The International Health Regulations (2005) list risk communication as one of eight core capacities that WHO Member States need to build and sustain as part of a global agreement to strengthen national and global systems to detect and respond to public health threats [5]. Risk communication and community engagement (RCCE) is an important approach for developing and disseminating accurate information, and it has been associated with more successful empowerment of affected local communities in disease outbreaks [6]. Experiences from the HIV, Ebola, Zika, and polio epidemics have demonstrated the cost to public health and health systems when rumors and misinformation are amplified in an environment where there is already a high level of distrust, which is aggravated by a poor public health communications response [7]. In a public health emergency or outbreak, existing service delivery may be disrupted and health authorities may not yet know the facts and have adequate evidence; this can lead to an information void, causing confusion and anxiety in the affected population [8]. If information voids are not responded to with high-quality health information, they can quickly be filled with misinformation and disinformation. Pieces of information of unknown validity can be benign and transient, or they can be false, causing damage if they affect individual and community decision-making. Rumors can be detrimental to health, especially in emergencies and crisis situations [4]. Rumors, unlike misinformation or disinformation, may be found to be true, and they can be either persistent and long-standing or evolve quickly after an acute event [4].

Overall, health emergencies give rise to information overload, which has been shown to influence people’s behavior, risk perception, and protective actions during health emergencies [9] and subsequently give rise to information avoidance. In emergencies, affected individuals and populations may have difficulty processing complex information and may retain only some of the early information they receive. In such circumstances, rumors can propagate quickly, challenging emergency responses that rely on the affected population to follow accurate health advice and enacting behaviors to protect individual and community health [8].

Although rumors and health misinformation have been around for as long as diseases, today’s environment is different. The COVID-19 infodemic has been an unprecedented challenge because we are experiencing an epidemic in a digitized globalized society. Digital tools and technologies have not only changed the way we communicate but have also changed our lives, altering the way we live, work, interact, and build our social identities and sense of community. For example, rumors and information have travelled across borders very quickly and influenced traditional media news cycles and coverage, emotive misinformation travels much more quickly across the digital media than fact-based health information, and epidemic control decisions or controversy in one country can cause debate and comparison with responses in other countries [9].

This infodemic has placed strain not just on how to communicate the evolving scientific knowledge but also on how public health authorities can implement a nimbler pandemic response that addresses the needs and concerns of local communities. During the COVID-19 response, health authorities have faced full-on the changed information and communication ecosystem [10] and its challenges, such as:

- Computational amplification of polarizing messages over factual ones, and use of bots and cyborgs to manipulate the outcome of online petitions, change search engine results, and boost certain messages on social media;
- Widespread microtargeting of social media users that is enabled by the social media and search engine platform business models, putting individuals into their own personalized “information bubbles”;
- Changed practices in TV and radio newsrooms that enable dissemination and amplification of poor-quality information that originates online;
- Weakened local media and collapse of local journalism, which have enabled mis- and disinformation to take hold.

In response to the infodemic, health authorities have needed to build partnerships beyond their usual networks—with fact-checkers; broader groups of media and journalists; social media, search engines, and digital interaction platforms; community organizations; civil society; and others. However, there is still room for improvement based on experience from the COVID-19 response. For example, although fact-checking organizations are relatively mature worldwide, half of them do not work with health professionals when fact-checking and debunking health-related claims, leaving room for better collaboration with health authorities and medical associations [11]. Moreover, whereas communication campaigns can raise the visibility of a set of messages, they are often not effective at debunking false claims, which require more quantitative and qualitative pretesting of messages; also, they must respond to questions, concerns, and narratives that are currently capturing people’s attention in a specific geographical area or a vulnerable community [12]. Mis-, dis-, and malinformation (also referred to as *information disorder*) are major and growing challenges, not only for emergency response but also for other societal actions [10].

Because of these challenges, the COVID-19 infodemic is not only a communication challenge but a challenge for the whole information ecosystem. Already at the beginning of the COVID-19 pandemic in April 2020, the WHO had crowdsourced a framework for managing infodemics that calls for whole-of-society involvement and response [3]. This framework recognized that in digitized society, the harmful effects of the infodemic cannot be managed through the prevailing approaches to communication, community engagement, and messaging alone. Infodemic response must take into account the information ecosystem, the ways we interact within the information ecosystem, and how information affects our health behavior. Consequently, this dynamic environment requires interventions across multiple levels, such as individual, community, medium, platform, policy, and others. The WHO infodemic management framework called for a multidisciplinary research agenda that informs the use of evidence-based interventions and surveillance across all phases of an epidemic [13], which led to the convening of this technical conference.

Between June and October 2020, the WHO Information Network for Epidemics (EPI-WIN) organized a global online technical conference followed by an asynchronous expert review exercise to develop a public health research agenda for infodemic management [3,13-17]. This transdisciplinary scientific consultation and review gathered infodemic insights and approaches from a wide range of relevant fields to inform and expand frameworks in infodemiology. Along with strengthening the foundations of an expanding infodemiology discipline [18] and creating the research agenda to direct focus and investment toward this emerging field, other aims of the conference were to improve understanding of the multidisciplinary nature of infodemic management; identify current examples and tools to understand, measure, and control infodemics; and establish a community of practice and research, preparing the ground for sustainable, long-term practices for responding to infodemics. The full conference report is available on the WHO website [17]. This paper summarizes the methods and results of the research agenda and the development of the research questions.

## Methods

### Overview

The research question prioritization exercise was designed in line with the WHO research agenda development guide for staff [19]. Held in the context of the COVID-19 pandemic and with travel restrictions in place, the consultation necessarily took place online via videoconference. The virtual discussions took place over 8 meeting days during 4 weeks in June and July 2020, and they resulted in a research agenda frame and a list of priority research questions. This was followed by asynchronous email communication from August to October 2020, during which participants were led through a structured expert opinion exercise to review and prioritize research questions within the set research agenda frame. Institutional Review Board review was not sought because the work described in this paper was based on observation of discussions at the conference, and it focused on the synthesis of expert opinion following the Chatham House Rule [20]. No personal information was collected from the participating experts.

### Format of the Virtual Conference

The 110 invited participants represented over 35 countries across 19 time zones, with a 56% to 44% gender split in favor of women (62 female, 48 male). They were academics selected by the organizers for the relevance of their publication record in the past two years for the purpose of this consultation, or practitioners who were working in pandemic response. A total of 60 additional invited academics were not available to participate. The conference participants represented 20 different academic and professional fields, such as digital health, computer science, communications and graphic design, media studies and journalism, history, applied mathematics, information science, data science and computational social sciences, complexity science, social and behavioral sciences, ethics, governance, marketing, and user experience and design; they were joined by colleagues from the fields of risk communication and community engagement, epidemiology, and public health, as well as by global public health implementing partners. Conflicts of interest were reviewed in accordance with WHO procedures for the management of declaration of interest for expert consultations [21]. The conference and follow-up communication were supported by a team of 49 organizers.

The meetings took the format of plenary sessions at the beginning and end of the conference and an in-between working session with four discussion sprints. Each participant was engaged in the meeting process for 18 hours (10 hours in plenary and 8 hours in topic discussions). Participants were split into four teams, grouping by similar time zone location but ensuring academic and practitioner diversity of the teams. Each team met four times for 2-hour “sprint sessions” of intense discussion on one of four topics, led by dedicated “topic masters” (scientific facilitators). The topic masters were scientists established in their scientific disciplines; 7 were academics employed by universities, and 1 was a WHO staff member with an academic affiliation. As the teams rotated from topic to topic, the topic masters facilitated discussions to collect insights from the discussion and validate expert opinion they had collected from discussion with preceding teams. By the end of the process, each team had discussed each topic, and each topic was discussed with four teams in an additive fashion—a total of 32 sprint hours of expert discussion.

The discussion sprints were oriented around four topics that mirror the epidemiological method for outbreak detection and management across the phases of the epidemic curve, enabling the actions of “preparing, monitoring, detecting, intervening, strengthening, and enabling” infodemic management. The topics were (1) how to measure and monitor digital and physical information environments; (2) how information originates and spreads; (3) how information affects individuals and populations; and (4) what interventions work to protect and mitigate against mis- and disinformation. By the end of the working session, a frame for a research agenda emerged based on the feedback from all the team discussions, seeded with draft research questions that were identified by the discussion facilitators.

In addition, the facilitator leaders of each of the four discussion streams at the conference summarized the discussions they had with all four teams of participants. Their reports summarized discussions about the main suggested research questions for the research agenda as well as enablers and challenges to researching them. This initial collected set of research questions became the basis for the follow-up process after the conference.

### Asynchronous Expert Ideation and Prioritization Exercise

After the virtual conference, the same participants were led through a 3-month asynchronous structured exercise that aimed to collect and rank research questions and to guide the participants toward a refined research agenda. In the exercise, structured expert judgment was collected through an adapted Delphi consultation using the Investigate Discuss Estimate Aggregate (IDEA) protocol [22]. The method involved asking the participants to devise and submit research questions that were relevant to the topic, answerable in the short or medium term, ideally capable of producing knowledge that could be put to use in the short or medium term, and focused on scope (ie, an answer to the research question should be provided in a single academic paper). They were also asked to focus on what would be scientifically feasible to answer and what had an expected public health benefit. To improve the reach beyond the pool of conference participants, each expert could invite up to two additional experts, based on their expertise and the value of their potential contributions. In total, 38 experts submitted additional research questions to the pool of candidate research questions and the following ranking survey. To maximize transparency in the categorization, experts could themselves choose which category or subcategory to submit a research question to. To identify potential gaps in the overall research agenda, the survey included open-ended questions.

A list of candidate research questions was built by combining the questions that were proposed by the topic facilitators based on the discussions at the conference and those that were collected through the survey round after the conference. The collected candidate research questions were assessed for topic overlap and scope, and they were edited and merged for clarity by three reviewers. The three experts were present in the discussions during the conference and are coauthors of this paper. Two are staff members of health authorities, and one is an academic. This reduced the questions to a consolidated list that was used in the research question ranking exercise.

The questions in the consolidated list were then anonymously scored and ranked through another exercise using the LimeSurvey platform [23]. There, participating experts were asked to rank the research questions based on two dimensions: public health impact and feasibility. These two ranking indicators were selected to point the agenda to evidence that can inform COVID-19 infodemic response quickly or with high impact, anticipating its importance in light of pandemic fatigue and the protracted use of public health and social measures to manage the pandemic, as well as ahead of the eventual introduction of COVID-19 vaccines. Public health impact was assessed through the question: Can this research lead individuals or communities to take healthy actions or help them understand why and how they do not take healthy actions? Research questions that could lead to meaningful change or adaptation of behaviors would be considered more impactful. Experts were asked to rate each question on a 5-point Likert scale (1, very low impact; 2, minor impact; 3, moderate impact; 4, high impact; 5, very high impact). Feasibility was assessed through answering the question “Can you think of a research project that would answer this specific question in a set number of months?” The faster the research project could be initiated and deliver results, the higher its feasibility and usefulness for the COVID-19 pandemic response. Experts were asked to rate this question on a 5-point Likert scale (1, 3 months; 2, 6 months; 3, 12 months; 4, 18 months; 5, 24+ months, based on emergency response planning time periods). A research question was considered high priority when it scored above 3 on impact and below 3 on feasibility.

To reduce potential survey fatigue and to avoid systematic missingness in the rankings (ie, due to respondents ranking only the first few questions within each stream), the order of the research questions to rank was randomized within each research stream. The randomization was conducted via the LimeSurvey platform. Following the ranking exercise, four experts reviewed the questions that fell outside the prioritization area—below the 3.5 consensus impact rank and with feasibility of >1 year. The four experts were three researchers that had previously reviewed the submitted research questions, with an additional staff member of a health authority who was not a coauthor on this paper. The fourth health authority staff member was added because the research agenda questions were meant to be feasible in a short time frame or highly important to the health authority response to the infodemic. The experts reviewed the questions outside the cutoff and, on consensus, they identified prerequisites or parts of these research questions that could be delivered with quicker feasibility and high public health impact. These research components were added to the research agenda as research questions.

**Figure 1:**
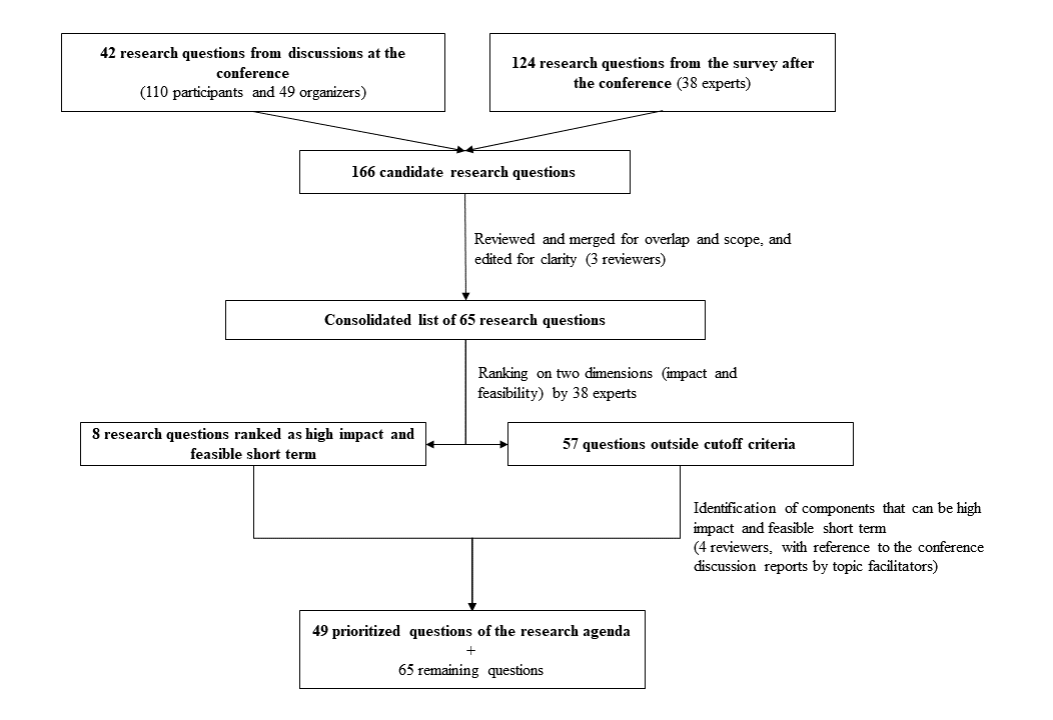
Refinement of the research questions through the structured expert judgment process.

This exercise reduced the number of questions to a shortlist of top priority and second tier priority questions per work steam, totaling 49 priority research questions. The remaining questions that were part of the exercise and did not make the prioritization cutoff were retained for reference, and they can be used for future reviews. The results of the recursive refinement of research questions through structured expert judgment exercise are summarized in Figure 1.

## Results

### Themes That Emerged During the Discussion Sprints

The discussion at the virtual conference reflected the complexity of the information ecosystem and the way it influences the strategies for managing the COVID-19 infodemic and other infodemics to support health behaviors and the management of epidemic risk. Several themes surfaced in the topic discussion sprints, as follows:

A common theme across discussions was that it is necessary to identify reproducible patterns and crossdisciplinary metrics for the science of infodemiology. Because access to full data sets from social media is rare and they do not represent the engagement of all populations, and because metrics vary from platform to platform, it is difficult to produce generalizable or comparable results. Mathematical modeling, such as epidemiological modeling, does not necessarily take human behavior into account, which can limit its efficacy to predict future human behavior and the impact thereof on an outbreak; however, modeling can aid the development of hypotheses for how information/infection flows, how networks might respond, and how interventions should be designed to test them. There are also limits to applying the epidemiological framework as a way to monitor and measure spread, especially if we assume that the unit we are working with is information instead of a virus, because viruses do not have an agenda and they infect opportunistically. Detangling the differences between rumors, misinformation, and disinformation requires a common taxonomy of information classification, some of which may be labelled as more harmful or less harmful. This could inform identification of the “tipping points” or when action needs to be taken to address more harmful misinformation by offering a more tailored and effective response.

Although it is important to describe the flow of health information, there needs to be a balance between a system-level understanding that “washes over” details and a case-study understanding that captures details but may miss the “bigger picture.” Substantial amounts of social and behavioral and health data are available; however, determining which data sources and types of analyses would improve a response needs a clearer definition. The degree of detail is needed to understand the infodemic while balancing privacy and ethical concerns, and managing limited analytic capacity in short time frames should be discussed. Amid a pandemic, speed is of the essence, and balancing rapid data collection and analysis methods with the desire for rigor may mean prioritizing specific kinds of data for short-term operational use versus longer-term, longitudinal trend analysis and use. Understanding the diffusion of information through certain networks may require other data collection approaches and discussion of how closed messaging apps and offline networks challenge this.

One area of research that needs further study is the extent to which offline behavior is being influenced by online behavior (and vice versa). There is limited research on how exposure to information or misinformation affects behavior because behavioral processes can be quite complex. Amid a crisis, people might use cognitive shortcuts and rely on the first information they hear; also, they may be less adept at processing more complex information. At the same time, there is little known about the longitudinal effects of the exposure to false claims that may not seem harmful at any one point in time but could have a cumulative harmful effect over time. In addition, when misinformation is easy to spread, this can create a harmful mixture. Anecdotal evidence suggests that people can exhibit negative health behaviors because of misinformation they heard during the COVID-19 outbreak; however, we need better measures of how knowledge connects to intent and behavior, both online and offline. For example, does increased exposure to misinformation make it more likely that someone will exhibit a behavior that is detrimental to their health? Further research is needed to develop better monitoring metrics, in addition to consolidated and validated indicators that predict behaviors or serve as proxies for specific behaviors.

The participants also emphasized that there is an interplay between information ecosystem actors and the resilience of communities and individuals. It was agreed that trust is a key element of building resilient communities. This leads to the need to establish and maintain trustworthy information sources. Some work must be done to identify these sources of information and to ensure easy and equal access. The discussions also highlighted the urgent need to empower communities to manage infodemics and build resilient communities through co-designed interventions. This would be made possible by understanding the context in which infodemics occur and spray. Community engagement goes along with building self-efficacy and self-capability through practice. It should focus on the “middle ground,” as in, the majority of “silent lurkers”—those who have not yet formed strong opinions. Besides individuals, communities, and states, members of the private sector should be regarded as actors. Internet platforms can be active vectors or targets of campaigns and can also be influential members of communities.

When considering long-term interventions, critical thinking and literacy (eg, health, information, digital, and media literacies) play important roles as a basis for interventions to address infodemics. Health literacy is a major topic in health communication research and practice. It includes critical literacy as the ability to evaluate and apply health information, and it is considered a major asset in managing an infodemic. Similarly, information, news, digital, and media literacies contribute to individuals’ ability to distinguish high- from low-quality information, especially online, and to the ability to improve their offline lives through digital technology use. Research into each type of literacy has developed in isolation, and questions remain on how to empower populations to think critically; what normative models of thinking are most appropriate for an infodemic; who is responsible for building literacy; and how literacy efforts can be integrated into existing societal systems (eg, school education) and be adapted to reach populations outside of the traditional educational settings.

To help prioritize interventions and actions, it is also necessary to identify priority populations based on key vulnerabilities. Population studies need to be conducted to identify specific individuals and groups of individuals who are at the greatest risk of not being able to critically assess misinformation and of spreading it. This approach should include studying people’s perceptions, beliefs, and knowledge, as well as the barriers and facilitators that can affect the access to and evaluation of credible health information as well as its use in offline life. Additionally, the alignment of information vulnerabilities with disease vulnerabilities should be considered.

### Public Health Research Agenda for Managing Infodemics

**Figure 2:**
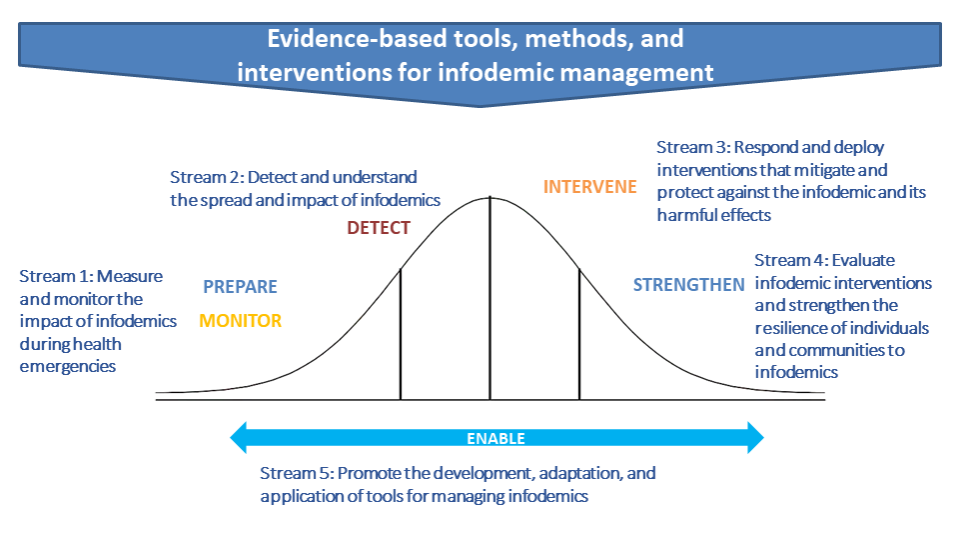
The frame of the research agenda mapped onto the phases of epidemic preparedness and response.

In addition to reviewing the current evidence and research gaps across different scientific fields, the conference participants sought to identify a research frame that could structure a public health research agenda for infodemic management. The aforementioned themes that emerged converged to broader landscaping of research gaps (eg, the need for better monitoring and metrics; localized and system-level characterization of infodemics; and understanding the components of the information ecosystem, individuals, communities, states, and private social media platforms). The themes also focused on some specific areas of knowledge gaps or promising interventions (eg, understanding the linkage between online and offline behavior; the roles of critical thinking and health literacy; and identification of priority populations). An agenda for future research should not only aim to fill gaps in the existing evidence base but, at high priority, also to link research evidence to public health practice. Therefore, the conference participants agreed to establish the research agenda frame analogous to the lens of epidemic management and to fit the identified themes, issues, and gaps to this epidemiological frame (see Figure 2). The framework’s streams were built on the activities of a health authority that supports outbreak response along the phases of an epidemic curve [24]—prepare and monitor, detect, intervene, strengthen, and enable infodemic response, as outlined below and in Table 1:

- Stream 1 supports the preparedness and monitoring of infodemics through measurement and monitoring of impact of infodemics. Standardized metrics and measurement tools can help characterize infodemics online and offline, identify absence of information where misinformation can gain more traction, and help recognize tipping points when detailed investigations need to take place. Last, evaluation of infodemic management interventions needs more elaboration.
- Stream 2 addresses the need to detect and understand the spread and impact of infodemics. In the context of infodemics, communities and vulnerable groups are no longer defined only in terms of geographies but can also be formed through shared values, goals, or motivations. Development of interventions therefore needs localized contextualized understanding of the infodemic, how misinformation affects behaviors in vulnerable groups, and understanding of the ethical and regulatory approaches needed to mitigate the spread of misinformation.
- Stream 3 addresses the response and deployment of interventions that protect and mitigate the infodemic and its harmful effects. Thinking about implementation of interventions needs to be built into the infodemic management activities and research so that the research is linked to what health authorities need to respond. To achieve this, behavior/change models relevant to infodemic management need to be developed, and interventions need to be designed.
- Stream 4 aims at research that strengthens infodemic management by development of common frames to improve intervention development and programmatic response to infodemics. Using the continuum of community engagement, local cultural context, and building resilience to infodemics and misinformation at individual, community, platform, and societal levels are addressed.
- Stream 5 supports the overarching aim to strengthen infodemic management practice by enhancing transferability of lessons learned and evidence-based interventions between contexts, countries, and infodemics. The information ecosystem and socioeconomic determinants of access and use of health information differ across countries; we therefore need to understand how interventions can be successfully transferred across countries and what impact they will have in other settings.

**Table 1:** Framework of the public health research agenda for managing infodemics.

Stream	Subtopics
Stream 1: Measure and monitor the impact of infodemics during health emergencies	1.1. Standardize taxonomies and classifications 1.2. Develop new metrics to measure and quantify infodemics 1.3. Analyze and triangulate data from multiple sources 1.4. Improve evaluation approaches for infodemic interventions
Stream 2: Detect and understand the spread and impact of infodemics	2.1. Understand how information originates, evolves, and spreads on different platforms and channels 2.2. Assess the role of actors, influencers, platforms, and channels 2.3. Understand how misinformation affects behavior in different populations 2.4. Develop regulatory and ethical principles to mitigate the spread and propagation of harmful health information
Stream 3: Respond and deploy interventions that mitigate and protect against the infodemic and its harmful effects	3.1 Design a behavioral/change model applicable to infodemic management 3.2. Design interventions for different levels of action to mitigate the infodemics
Stream 4: Evaluate infodemic interventions and strengthen the resilience of individuals and communities to infodemics	4.1. Develop interventions that address individual, community, cultural and societal-level factors affecting trust and resilience to misinformation 4.2. Understand and learn from how misinformation has affected behavior among different populations and in different contexts for specific infodemics 4.3. Identify factors associated with successful infodemic management by health authorities, the media, civil society, the private sector, and other stakeholders
Stream 5: Promote the development, adaptation, and application of tools for managing infodemics	5.1. Use implementation research evidence in program improvement and policy development 5.2. Promote evidence-based interventions and approaches among countries 5.3. Improve effectiveness and response times to the infodemic during acute health events

At the conclusion of the conference, 42 research questions were collected from the topic discussions, as curated by the scientific topic facilitators. During the follow-up research question generation exercise, 38 experts submitted an additional 124 research questions across 5 research streams and 16 subcategories. This added up to 166 candidate research questions. These research questions were reviewed and merged for repetition, overlap, and scope; they were then edited for clarity by three reviewers. Suggestions that were not formulated as research questions were excluded. This review identified a high degree of overlap and repetition, pointing to a saturation of topics submitted for the ranking exercise. It resulted in a consolidated list of 65 questions that were subjected to the ranking exercise.

**Figure 3:**
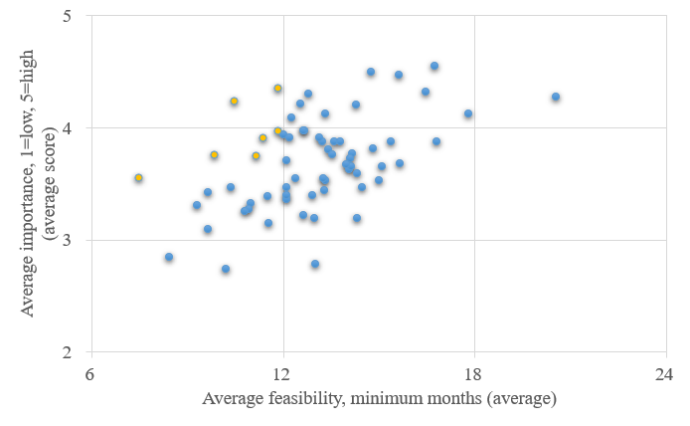
Ranking of the surveyed research questions across two indicators: public health impact and feasibility. Research questions that were within the cutoff limit of minimum 3.5 impact and less than 12 months feasibility are marked in yellow. Questions that were ranked outside the cutoff limits were reviewed and broken into additional smaller component questions that were of high value.

The research questions to be ranked were evenly distributed, with at least 10 questions included for ranking in each of the five research streams (18 in stream 1, 16 in stream 2, 10 each in streams 3 and 4, and 11 in stream 5). The ranking exercise results for these questions are depicted in Figure 3.

Following the ranking exercise, four experts reviewed the results. Based on the ranking exercise, only 8 research questions covering streams 1, 2, and 3 were prioritized with a consensus rank greater than 3.5 and feasibility of <1 year. Therefore, the experts reviewed the 8 ranked questions and the remaining 57 questions that fell outside the cutoff limit. Based on their expert judgment and guidance from the reports of topic discussions during the conference, they identified precursor questions or components of these research questions that could be delivered with quicker feasibility or higher public health impact. The experts worked with the goal to use the questions and the feedback collected in the ranking exercise and used them as a guide to formulate research questions that could be their precursors. They worked on consensus and formulated the final list of 49 research questions, and they retained the additional 65 questions for future reference.

Expert review of the results of the ranking exercise identified 3 top research questions per work stream, resulting in a list of 15 top priority research questions for the public health research agenda for infodemic management (Table 2). Further, a second tier of important research questions was set for each subtopic, totaling 34 questions. Multimedia Appendix 1 shows the prioritized research questions and agenda in more detail, as well as the additional 65 research questions that were not identified as a high priority in the short term. These can be used to map further evidence gaps on the topics and for reference and guidance in subsequent research agenda reviews [17].

**Table 2:** Top 15 research questions across five streams of the research agenda.

Stream	Top 3 questions per stream
Stream 1: Measure and monitor the impact of infodemics during health emergencies	What are ways to score health-related misinformation according to its potential for harm (to people’s health and behaviors; social cohesion; trust in health service delivery, government, communities, media; etc)? How do the infodemic curve and measures of spread and impact change over time during the phases of a disease outbreak? What are the potential indicators or their proxies for measuring trust, resilience, behavior change, exposure to misinformation, susceptibility to misinformation, social cohesion, depth of community engagement, etc?
Stream 2: Detect and understand the spread and impact of infodemics	How does misinformation mutate, adapt, or become remixed between infodemics and within infodemics? What are the strategies used to reduce misinformation’s potential harmfulness in closed networks (online and offline)? How do different types of health misinformation affect online and offline behavior, and what are some measures that can help forecast the impact of the health misinformation types on behavior?
Stream 3: Respond and deploy interventions that mitigate and protect against the infodemic and its harmful effects	What behavioral or process models can inform the development of an infodemic strategy and measure its impact at the individual, community, platform, or societal level? What are the promising interventions at the societal/community/individual/health system levels to address and mitigate health misinformation? What types of participatory or human-centered design approaches can be used to produce more tailored and effective infodemic management interventions?
Stream 4: Evaluate infodemic interventions and strengthen the resilience of individuals and communities to infodemics	How might we define and measure the gradient of community engagement, trust, and empowerment at the individual and community levels as they relate to infodemic management and reduction of harm from health misinformation? What are the sociobehavioral, mental heuristics, and design hierarchies that need to be considered when developing an intervention at the individual and community level? What are the “best buy interventions” to be used by different types of actors in society to maximize the impact on the infodemic at a lower marginal cost?
Stream 5: Promote the development, adaptation, and application of tools for managing infodemics	What considerations should be included in the assessment of risk, harms, and opportunities during the design and implementation of research and infodemic management interventions? What would a readiness assessment look like for infodemic preparedness for a new COVID-19 health intervention? What recommendations can be made to update the International Health Regulations to incorporate infodemic management more strongly as a core capacity of Member States?

## Discussion

### Principal Findings

Throughout the consultation, the discussions built progressively; participants shared a wealth of experience, discussed the challenges and benefits of various approaches, clarified the initial topics, and ultimately achieved a high degree of consensus about the needs that the research agenda would have to meet. The overarching conclusion was the need to complete and implement the research agenda along with the framework for action [3,13]. The takeaway action points from the conference are as follows.

Information, misinformation, and public health are intertwined by nature: the WHO has dealt with issues at the intersection of misinformation, trust, and demand for health services since it was founded. Lessons from this experience have led to evolved epidemic response methods, tools, and the global response community over time. The WHO and other partners who work in the fields of public health communication, risk communication, and community engagement have been challenged by the scale of the COVID-19 infodemic, which has been amplified by the global digitized information ecosystem. In a new century, addressing new types of outbreaks requires innovative and precise public health tools [25]. Different populations have different information needs, channels, and barriers. Evidence-based interventions are needed at all levels—for individuals, communities, platforms, health systems, and societies—to reduce the transmission and impact of the disease. Coordination, connection, and integration across disciplines and sectors must be central to expanding the scientific discipline of infodemiology. Rapid application of the science during the COVID-19 pandemic needs (1) sustained integration across the various disciplines of research; (2) integration between research, practice, and lived experience; and (3) inclusion of representation and voices from different sociocultural contexts in practice and lived experience.

At the same time, infodemic management must broaden its tools beyond communication and consider all components of the information ecosystem [7,26,27]. Because the information ecosystem spans both online and offline environments, it is more difficult to detect and respond to the infodemic in communities as well as to work proactively to build resilience and a healthier information ecosystem overall. Media, policy makers, and the private sector influence the information ecosystem where individuals, interest groups, civil society, academia, fact-checkers, and others also interact. Partnerships between health authorities, fact-checkers, media organizations, and other global public health partners, such as the Africa Infodemic Response Alliance [28] are critical to effectively promoting high quality health information and countering health misinformation at local level. The RCCE collective service [29] was started in June 2020 to concentrate the RCCE capacities across global RCCE partners. Strengthened partnerships at local levels are also needed to focus on community engagement in offline communities. On the other hand, regulatory interventions could help standardize access to social platform data, ensuring that the data we do have access to is comprehensive and regular. Access to regular and better data/metadata would increase accountability for how a healthier online information ecosystem is built. Based on data availability, this access could also facilitate the design of research and interventions to give us a better understanding of which interventions work online and the conducting of independent analyses of information provided by the platforms.

Addressing the harms of infodemics is important because they impact health behaviors and are barriers to healthy life and well-being. It is important to better understand proactive strategies that apply social inoculation theory or literacies theory in building resilience. Developing health literacy is critical and includes access to health services literacy, and it is dependent on digital nativity/technological skills, access to information, and media literacy/interrogative skills. The reasons why mis- and disinformation spread are complex; therefore, it is important not to reduce that complexity by framing infodemic management as simply a battle against misinformation [7,30-34]. It is equally important to reinforce and accelerate health-enhancing behaviors and generate information to help people develop resilience to information disorder. In the long term, this will help people build trust, make informed decisions, and access essential health services, and it will have impact far beyond the COVID-19 pandemic.

Given the urgency of pandemic response, the new transdisciplinary practice will have to learn from practice and iteration even as it develops, reporting experience gained through implementation to provide more evidence on what works and what does not [35]. Ultimately, health authorities need to identify and allocate the necessary capacity to manage infodemics. This is a programmatic and process issue. Once that capacity is in place, decision-makers and the private sector need to develop, validate, implement, and adapt tools for infodemic management during acute public health events in culturally and contextually appropriate ways. The issue of connecting this evolving practice and research is not trivial: the community that implements the research agenda must be, and must remain, a community of practice *and* research that prioritizes questions to inform operations and improve contemporary practice, foregrounding the pragmatic needs of people in the field and on the ground.

We also need to think about how to build systems for social listening, signal detection, and the analysis of infodemics and misinformation. For example, investment is needed to develop a shared, open reference database for characterizing misinformation (including examples) to identify appropriate interventions and when and how to deploy them [36]. Effectively, the content would be re-contextualized to enable characterization and use in the analysis. This database could include different types and sources of misinformation, the intent of those creating or sharing misinformation, the degree of inaccuracy (based on the level of expert consensus and scientific evidence that exists), its impact on attitudes or behaviors, the likely audience, its virality, or its alignment with politics. This reference database could be populated with specific examples of misinformation that fall into each domain, which could then be aligned with interventions based on best practices shown to be effective for that type of misinformation. Such a reference database would help to answer questions about the differences between the content people will merely share online and the content that will affect their decision-making and offline behavior. It would also aid the investigation of whether we can use content characteristics to predict the likelihood of spreading a piece of content in different ways.

The community of research and practice could also develop and use a shared “living systematic review” for interventions measured in terms of effectiveness on a set range of criteria, strength of evidence, generalizability, and likely contexts for application. Interventions across disciplines could be collected, with a rubric describing the outcomes against which the intervention has been tested, its generalizability or application to specific populations, the contexts in which it has been tested, its feasibility and costs, and the confidence in the findings. This could include determining the consistent metrics appropriate to evaluating the success of an intervention to prioritize efforts. However, it is unlikely that only one intervention will be successful; a toolkit of different approaches will likely be appropriate. This living systematic review could be aligned with the misinformation reference database to identify gaps where good evidence-based research does not exist to address certain types of misinformation.

### Conclusions

The resulting public health research agenda for infodemic management will be maintained on the WHO website as a living document; its implementation and priorities will be reviewed and adjusted regularly.

Infodemics impact people, including health professionals, globally. Although infodemics are not new, addressing them in the new digitized society is a different and centrally important challenge in responding to the COVID-19 pandemic as well as future pandemics. The research agenda that emerged from this consultation crystallizes themes that can inform initiatives to build the foundations of effective infodemic management in all countries. The main target audience for these research questions are researchers and practitioners. They will also be of interest to public health experts, nongovernmental organizations, the media, and other stakeholders.

There is a large gap between infodemiology research and evidence that has been generated by the academic disciplines and the response to the infodemics. Tools and interventions that are grounded in this evidence are sorely needed by health authorities worldwide. This is partially because scientific disciplines have worked in a mostly disconnected fashion on addressing the challenge of information overload, communication, design, media studies, sociobehavioral factors, misinformation, and the ethics and regulation of the information ecosystem. The WHO and its Member States and partners must close this gap by developing and adopting evidence-based tools that are appropriate for their local contexts. This consultation and the previous infodemic management meeting [3,13] may have been among the first opportunities for many people working toward this goal to hear about the expertise and activities of others, and to frame the entirety of this activity within the problems of disease control and public health.

Following the conference, the WHO partnered with five scientific journals in a joint call for papers for special issues on infodemiology [37], two of which have already been published [38,39]. The WHO EPI-WIN team has used the outcomes of this conference as the input in the third and fourth WHO infodemic management conferences [40,41] and the upcoming fifth WHO infodemic management conference, which will focus on the development of measurements and metrics for infodemic management. The research gaps that were identified have also guided the WHO in the review of the COVID-19 research blueprint [42] and in the development of partnerships that foster filling of research gaps and for translation of evidence into use by health authorities and other partners [28,43]. The WHO has also applied evidence and infoveillance methods to inform its own work and contribute to the development of metrics for health authorities [18,44,45].

The challenge of a novel pandemic pathogen intertwined with an infodemic is a double burden that demands action-oriented research to inform public health response. The new research agenda will strengthen the scientific understanding of how infodemics impact populations and their health, but it will also serve as a basis for action and learning for future preparedness, strengthened through cross-sectoral pilot projects and continuous after-action reviews to build capacity. After the acute phase of the COVID-19 pandemic, we need to shift the focus to strengthening longer-term capacities and advocating for the inclusion of new tools and indicators. When applied to acute health events, the evolving research discipline of infodemiology can provide crucial evidence and facilitate multidisciplinary expertise and coordination.

## Appendix

Multimedia Appendix 1 Public health research agenda for managing infodemics (all prioritized and collected research questions).

## Abbreviations

EPI-WIN: World Health Organization Information Network for Epidemics
IDEA: Investigate Discuss Estimate Aggregate protocol
RCCE: risk communication and community engagement
WHO: World Health Organization

## References

[1] COVID-19 dashboard. World Health Organization.

[2] WHO Director-General's speech at the Munich Security Conference, 15 February 2020. World Health Organization.

[3] Tangcharoensathien V, Calleja N, Nguyen T, Purnat T, D'Agostino M, Garcia-Saiso S, Landry M, Rashidian A, Hamilton C, AbdAllah A, Ghiga I, Hill A, Hougendobler D, van Andel J, Nunn M, Brooks I, Sacco PL, De Domenico M, Mai P, Gruzd A, Alaphilippe A, Briand S (2020). Framework for managing the COVID-19 infodemic: methods and results of an online, crowdsourced WHO technical consultation. J Med Internet Res.

[4] Kou Y, Gui X, Chen Y, Pine K (2017). Conspiracy talk on social media: collective sensemaking during a public health crisis. Proc ACM Hum-Comput Interact.

[5] (2008). International Health Regulations (2005) Second Edition. World Health Organization.

[6] Walker B, Adukwu E (2020). The 2013-2016 Ebola epidemic: evaluating communication strategies between two affected countries in West Africa. Eur J Public Health.

[7] Vicol D, Tannous N, Belesiotis P, Tchakerian N, Stewart R Health Misinformation in Africa, Latin America and the UK: Impacts and Possible Solutions. Full Fact.

[8] Reynolds Barbara, W Seeger Matthew (2005). Crisis and emergency risk communication as an integrative model. J Health Commun.

[9] Brennen J, Simon F, Howard P, Nielsen R (2020). Types, sources, and claims of COVID-19 misinformation. Reuters Institute at University of Oxford.

[10] Wardle C, Derakhshan H (2017). Information Disorder: toward an interdisciplinary framework for research and policy making. Council of Europe.

[11] (2020). Health authorities and innovative collaborations across society to combat the infodemic. World Health Organization.

[12] (2020). Vaccine Misinformation Management Field Guide. UNICEF.

[13] (2020). An ad hoc WHO technical consultation managing the COVID-19 infodemic: call for action, 7-8 April 2020. World Health Organization.

[14] (2020). 1st WHO Infodemiology Conference. World Health Organization.

[15] (2020). Pre-conference: WHO Infodemiology Conference. World Health Organization.

[16] (2020). Post-conference: 1st WHO Infodemiology Conference. World Health Organization.

[17] (2021). WHO public health research agenda for managing infodemics. World Health Organization.

[18] Purnat T, Vacca P, Burzo S, Zecchin T, Wright A, Briand S, Nguyen T (2021). WHO digital intelligence analysis for tracking narratives and information voids in the COVID-19 infodemic. Studies in Health Technology and Informatics Volume 281: Public Health and Informatics.

[19] (2020). A systematic approach for undertaking a research priority-setting exercise: guidance for WHO staff. World Health Organization.

[20] Chatham House Rule. Chatham House.

[21] Declarations of interest. World Health Organization.

[22] Hemming V, Burgman MA, Hanea AM, McBride MF, Wintle BC (2017). A practical guide to structured expert elicitation using the IDEA protocol. Methods Ecol Evol.

[23] LimeSurvey.

[24] (2017). Emergency response framework (ERF), 2nd edition. World Health Organization.

[25] Dunn AG, Mandl KD, Coiera E (2018). Social media interventions for precision public health: promises and risks. NPJ Digit Med.

[26] Shane T, Noel P (2020). Data deficits: why we need to monitor the demand and supply of information in real time. 28 Sep 2020.

[27] Balog-Way DHP, McComas KA (2020). COVID-19: Reflections on trust, tradeoffs, and preparedness. J Risk Res.

[28] The Africa Infodemic Response Alliance. World Health Organization.

[29] The Collective Service: a new partnership for strengthening risk communication and community engagement in public health and humanitarian emergencies. World Health Organization.

[30] Tripodi F Searching for Alternative Facts: Analyzing Scriptural Inference in Conservative News Practices. Data & Society.

[31] Pool J, Fatehi F, Akhlaghpour S (2021). Infodemic, misinformation and disinformation in pandemics: scientific landscape and the road ahead for public health informatics research. Stud Health Technol Inform.

[32] Soroya SH, Farooq A, Mahmood K, Isoaho J, Zara S (2021). From information seeking to information avoidance: understanding the health information behavior during a global health crisis. Inf Process Manag.

[33] Tentolouris A, Ntanasis-Stathopoulos I, Vlachakis PK, Tsilimigras DI, Gavriatopoulou M, Dimopoulos MA (2021). COVID-19: time to flatten the infodemic curve. Clin Exp Med.

[34] Lewandowsky S, van der Linden S (2021). Countering misinformation and fake news through inoculation and prebunking. Eur Rev Soc Psychol.

[35] Purnat TD (2020). Building systems for respond to infodemics and build resilience to misinformation. LinkedIn.

[36] Dunn A, Steffens M, Dyda A, Mandl K (2021). Knowing when to act: a call for an open misinformation library to guide actionable surveillance. Big Data Soc.

[37] (2020). Joint call for papers - special issues on Infodemiology. World Health Organization.

[38] Gruzd A, De Domenico M, Sacco Pl, Briand S (2021). Studying the COVID-19 infodemic at scale. Big Data Soc.

[39] (2021). Special feature: infodemics and health security. Health Security.

[40] (2020). 3rd virtual global WHO Infodemic Management conference. World Health Organization.

[41] (2021). 4th virtual WHO Infodemic Management conference: advances in social listening for public health. World Health Organization.

[42] World Health Organization.

[43] Gesualdo F, Bucci LM, Rizzo C, Tozzi AE (2021). Digital tools, multidisciplinarity and innovation for communicating vaccine safety in the COVID-19 era. Hum Vaccin Immunother.

[44] Purnat TD, Vacca P, Czerniak C, Ball S, Burzo S, Zecchin T, Wright A, Bezbaruah S, Tanggol F, Dubé Ève, Labbé Fabienne, Dionne M, Lamichhane J, Mahajan A, Briand S, Nguyen T (2021). Infodemic signal detection during the COVID-19 pandemic: development of a methodology for identifying potential information voids in online conversations. JMIR Infodemiology.

[45] Purnat TD, Wilson H, Nguyen T, Briand S (2021). EARS - a WHO platform for AI-supported real-time online social listening of COVID-19 conversations. Stud Health Technol Inform.

