# Supplementary material for: A Public Health Research Agenda for Managing Infodemics: Methods and Results of the First WHO Infodemiology Conference
Source: JMIR Infodemiology. 2021 Sep 15;1(1):e30979. doi: 10.2196/30979 (PMC8448461; doi:10.2196/30979)
Supplement: Multimedia Appendix 1 [file infodemiology_v1i1e30979_app1.docx]

## Multimedia Appendix 1

## Public health research agenda for managing infodemics (all prioritised and collected research questions)

Top three priority research questions overall across all five streams:

1. What are ways to score health-related misinformation in regard to its potential for harm (to people’s health, unhealthy behaviors, social cohesion, mistrust in health service delivery, mistrust in government, mistrust in communities, mistrust in media, etc.)?
2. How do different types of health misinformation affect online and offline behavior and what are some measures that can help forecast impact of the health misinformation types on behavior?
3. What would a readiness assessment look like for infodemic preparedness for a new COVID-19 health intervention?

**The top ranked research questions within each workstream are marked in yellow and bolded.**

## Research stream 1: Measure and monitor the impact of infodemics during health emergencies

Standardized metrics and tools are needed to track the evolution of infodemics in the digital-physical information environment, between individuals, communities, society and the health system, using multidisciplinary approaches, including methods and approaches from Artificial intelligence, Natural Language Processing, and using structured and unstructured data (big data, ethnographic data, and similar).

Standardized metrics and tools are needed to track the evolution of infodemics among individuals, communities, societies and health systems, in both the digital and the physical information environments. This requires multidisciplinary approaches, methods and tools including those from the fields of artificial intelligence and natural language processing, and the use of structured and unstructured data (including but not limited to “big data” approaches and ethnographic data).

Infodemics do not only propagate in the digital world: they occur online and offline and can be very harmful to vulnerable non-networked populations as well. Information flows or behaves differently depending on what kind of network it is in. Standardized metrics and tools can help recognize tipping points for when detailed investigations need to take place.

It is also important to recognize situations where information is absent, because this is where misinformation can gain more traction. Damaging misinformation can manifest especially well in the absence of accurate, credible information from sources trusted by individuals and communities. Promoting accurate, actionable information must therefore be seen as a process, not an end state.

Top three questions within this stream:

- What are ways to score health-related misinformation in regards to its potential for harm (to people’s health, unhealthy behaviors, social cohesion, mistrust in health service delivery, mistrust in government, mistrust in communities, mistrust in media, etc.)?
- How do the infodemic curve and measures of spread and impact change over time during the phases of a disease outbreak?
- What are the potential indicators or their proxies for measuring trust, resilience, behavior change, exposure to misinformation, susceptibility to misinformation, social cohesion, depth of community engagement, and similar?
  1. **Standardize taxonomies and classifications**
  2. What are the classes of health-related misinformation and disinformation that can be used for standardized research analysis?
  3. What reference datasets and algorithms are needed to establish standardized measures of infodemic?
  4. How can we compare infodemics and how they manifest in different countries to detect and understand patterns?

**1.2 Develop new metrics to measure and quantify infodemics**

- 1. **What are ways to score health-related misinformation in regards to its potential for harm (to people’s health, unhealthy behaviors, social cohesion, mistrust in health service delivery, mistrust in government, mistrust in communities, mistrust in media, etc.)?**
  2. What social network analysis principles are most relevant to generate rapid insights for use in routine analysis and in acute emergencies to inform public health response?
  3. What attributes contribute to high self-efficacy in individuals to identify and address misinformation effectively?
  4. What methods from computational science, digital analytics and other fields are applicable to infodemic monitoring?

**1.3 Analyse and triangulate data from multiple sources**

- 1. How are the epidemic curve and infodemic impacts related?
  2. **How do the infodemic curve and measures of spread and impact change over time during the phases of a disease outbreak?**
  3. What are standards, methods and approaches that can be used to co-analyze different data types from disparate data sources related to use in infodemic management ?

**1.4 Improve evaluation approaches for infodemic interventions**

- 1. **What are potential indicators or their proxies for measuring trust, resilience, behavior change, exposure to misinformation, susceptibility to misinformation, social cohesion, depth of community engagement, and similar?**
  2. What role does participatory evaluation play in infodemic intervention implementation?

## Research stream 2: Detect and understand spread and impact of infodemics

A common approach is needed to understand how an overabundance of information and misinformation is spread and how it affects online and offline behavior in different populations.

A common approach is needed to understand how information and misinformation is spread and how it affects online and offline behavior in different populations.

Averaging data at national or regional level is not helpful when trying to understand how an infodemic impacts communities and individuals. Communities cannot be defined soly by physical boundaries: they can also consist of geographically disparate people with shared lifestyles, life stages, values, goals or motivations. Information and misinformation are usually shared peer to peer, and every infodemic is in this sense local. A localized contextual understanding of the infodemic is critical for the development of interventions, whether those contexts are geographic or otherwise in nature.

We currently do not know how a person’s digital behavior reflects their offline behavior. We also have a difficult time tracking misinformation through platforms and figuring out what and how much of this behavior is relevant to track and understand. Analysis is driven by the data that is available, which creates an abundance of information on some topics and blind spots in others.

Top three questions within this stream:

- How does misinformation mutate, adapt or get remixed between infodemics and within infodemics?
- What are the strategies used to reduce misinformation’s potential harmfulness in closed networks (online and offline)?
- How do different types of health misinformation affect online and offline behavior and what are some measures that can help forecast impact of health misinformation types on behavior?

**2.1 Understand how information originates, evolves and spreads on different platforms and channels**

- 1. What are the attributes and drivers within the information environment, communication tools, and platform design that encourage the sharing of misinformation?
  2. **How does misinformation mutate, adapt or get remixed between infodemics and within infodemics?**
  3. What are the elements for classifying content for its potential for amplification and organic spread online?
  4. What are the attributes that promote faster spread of misinformation across platforms/channels/community boundaries?

**2.2 Assess the role of actors (influencers), platforms and channels**

- 1. What are strategies that could be used on online communication platforms to reduce the spread of misinformation online?
  2. What are the best practices for engaging with influencers to address misinformation with unique and tailored content that has high potential to amplify further?
  3. **What are the strategies used to reduce misinformation’s potential harmfulness in closed networks (online and offline)?**
  4. What role do advocates, fence-sitters, lurkers/observers and opponents of accurate information play in the spread of misinformation?
  5. How do analysis and intervention options differ between health disinformation and health misinformation?

**2.3 Understand how misinformation affects behavior in different populations**

- 1. How does misinformation spread and affect marginalized, vulnerable and at-risk populations?
  2. How can health-related concerns be used or weaponised to target specific groups of people with disinformation and threaten health security and social cohesion?
  3. **How do different types of health misinformation affect online and offline behavior and what are some measures that can help forecast impact of health misinformation types on behavior?**

**2.4 Develop regulatory and ethical principles to mitigate the spread and propagation of harmful health information**

- 1. What policies or policy tools are available to health authorities that balance the need between propagating accurate information, curbing misinformation, and ensure human rights of access to information?

## Research Stream 3: Respond and deploy interventions that protect and mitigate the infodemic and its harmful effects

An evidence base is needed to identify interventions that are effective in different contexts, populations, cultures, and for different types of acute health events.

An evidence base is needed to identify interventions that are effective in different contexts and for different types of acute health events. Infodemiology must be based on efforts to understand which interventions work and which ones do not, and for which populations and types of misinformation. Implementation thinking must be factored into all infodemic management activities, to avoid the risk of doing harm by detaching research from the pragmatic priorities of health authorities’ responses.

Top three questions within this stream:

- What behavioral or process models can inform the development of an infodemic strategy and measure its impact at individual, community, platform or society level?
- What are promising interventions at the (societal/community/individual/health systems) to address and mitigate health misinformation?
- What types of participatory or human-centred design approaches can be used to produce more tailored and more effective infodemic management interventions?

**3.1 Design behavioral/change model applicable to infodemic management**

- 1. **What behavioral or process models can inform the development of an infodemic strategy and measure its impact at individual, community, platform or societal level?**

**3.2 Intervention design for different levels of action to mitigate the infodemics**

- 1. **What are promising interventions at the (societal/community/individual/health systems) to address and mitigate health misinformation?**
  2. **What types of participatory or human centred design approaches can be used to produce more tailored and more effective infodemic management interventions?**
  3. What types of infodemic management interventions are most appropriate to deploy rapidly in acute health events or emergencies?
  4. What are the attributes of interventions that are effective across different contexts, populations, cultures, and for different types of acute health events?
  5. What are the considerations for designing infodemic management interventions when balancing promoting access to accurate health information and protecting freedom of expression?

## Research Stream 4: Evaluate infodemic interventions and strengthen resilience of individuals and communities to infodemics

Common evaluation frames are needed to improve intervention development and programmatic response to infodemics.

Common evaluation frameworks are needed to improve the development of interventions and programmatic responses to infodemics. It is not sufficient simply to provide more and more information and hope that this changes behavior. People will often interpret information based on their culture, past experiences and environment. Empathy and good information design are therefore needed to develop interventions that meet the needs of individuals and communities—ideally developed in collaboration with them. Established top-down approaches will not be sufficient simply because they are established. Interventions are required from individuals and influencers who are trusted and those who represent values that communities accept and listen to. Building that trust and implementing it effectively takes time and consistent engagement.

Top three questions within this stream:

- How might we define and measure the gradient of community engagement, trust and empowerment at individual and community level in their relation to infodemic management and reduction of harm from health misinformation?
- What are the socio-behavioral and mental heuristics that need to be considered when developing an intervention at individual and community level?
- What are the “best buy interventions “ to be used by different types of actors of society, to maximize the impact on the infodemic at a lower marginal cost?

**4.1 Development of interventions that address individual, community, cultural and societal-level factors affecting trust and resilience to misinformation**

- 1. How might we define and measure resilience to misinformation at individual and community level?
  2. **How might we define and measure the gradient of community engagement, trust and empowerment at individual and community level in their relation to infodemic management and reduction of harm from health misinformation?**
  3. **What are the socio-behavioral and mental heuristics that need to be considered when developing an intervention at individual and community level?**
  4. What role does social inoculation play in developing resilience to misinformation in the context of infodemic?
  5. What principles from social marketing can be leveraged to build demand for accurate health information at individual and community level

**4.2 Understand and learn from how misinformation has affected behavior in different populations and contexts for specific infodemics**

- 1. What are the lessons learned from infectious diseases such as Ebola, Zika, and polio on how to effectively combat misinformation at community level that applies to COVID-19?
  2. What are the lessons learned about information environments and misinformation spread related to non-communicable diseases and risk factors such as cancer and smoking?

**4.3 Identify factors associated with successful infodemic management by the health authorities, media, civil society, private sector, and other stakeholders**

- 1. What are the roles, responsibilities, and possible spheres of influence on the infodemic by different types of actors of society?
  2. **What are the “best buy interventions “ to be used by different types of actors of society, to maximize the impact on the infodemic at lower marginal cost?**
  3. What are the factors of success for collaboration between the health authorities and factchecking organizations/media, civil society, private sector?

## Research stream 5: Promote the development, adaptation and application of tools for managing infodemics

There is a need to enhance transferability of lessons learned and evidence-based interventions among contexts, countries and infodemics.

The transferability of lessons and evidence-based interventions among contexts, countries and infodemics need to be enhanced. Although every country has experienced some degree of the infodemic related to COVID-19, this consultation made the need for a multidisciplinary approach obvious to address infodemics in specific contexts. Readiness assessment tools are needed to allow strategic oversight of local capacities and environments so that tools and interventions can be adapted accordingly. Different communities display variations in levels of literacy and education, relationships with technology and patterns in using it, levels of internet penetration and cost of access, and the ability to assess information critically. It is not possible to copy the same interventions into different countries and hope that they will always work in the same way. Tailored resources are critical, the one-size-fits-all approach is not helpful in getting the reach and uptake that is required.

Top three questions within this stream:

- What considerations should be included in the assessment of risk, harms, and opportunities during the design and implementation of research and infodemic management interventions?
- What would a readiness assessment look like for infodemic preparedness for a new COVID-19 health intervention?
- What recommendations can be made to update International Health Regulations to incorporate infodemic management more strongly as a core capacity of Member States?

**5.1 Using implementation research evidence in programme improvement and policy development**

- 1. **What considerations should be included in the assessment of risk, harms and opportunities during the design and implementation of research and infodemic management interventions?**
  2. How can we ensure that infodemic management research and interventions are not misused for non-public health purposes?
  3. How can we ensure that infodemic management research and interventions do not cause unintended harm or are misused to purposefully cause harm?

**5.2 Promote evidence-based interventions and approaches between countries**

- 1. **What would a readiness assessment look like for infodemic preparedness for a new COVID-19 health intervention?**
  2. What types of infodemic management interventions are more transferrable across countries, populations and health events?

**5.3 Improve response times and effectiveness of responses to infodemics during acute health events**

- 1. What standardized capacities should health authorities have to address an infodemic concurrent to a disease outbreak?
  2. What would a severity grading look like for rating infodemic threats and potential appropriate response options?
  3. **What recommendations can be made to update International Health Regulations to incorporate infodemic management more strongly as a core capacity of Member States?**

Other questions that were considered in the prioritisation exercise:

- How can artificial intelligence and machine learning help in exploiting multi-source or other data?
- How can metrics provide evidence of impact at multiple levels (institutional, societal and community-level, individual attitudes, individual behavior)?
- How can measurement and evaluation frameworks employed by communicators be applied for measuring and monitoring the spread of misinformation in epidemics?
- Based on previous experience, what would a taxonomy for mis-/disinformation and rumors look like for different public health emergencies and based on previous experiences?
- What are the distinctive and objective indicators that characterise an infodemic? What datasets can be used?
- What are the lessons learnt from past infodemics that can be applied to this one?
- Which are the predominant themes around which information/misinformation spread the fastest during outbreaks?
- How is the concept of infodemic understood by epistemic communities – local authorities and policy makers, researchers, clinicians, and media? Especially in low and middle income countries.
- What is the measurable threshold after which an outbreak of dis-/misinformation becomes an infodemic at the policy level? How can it be measured?
- How to characterize clearly and systematically what constitutes harmful health information, i.e. information with harmful consequences?
- How to monitor the impact of COVID-19 related misinformation in low- and middle-income countries? What media to look at, what tools to use?
- What populations are more susceptible and exposed to infodemics?
- What are the pathways and influences through which rumors and misinformation reach those who are not digitally connected?
- How do national and regional contexts relate to the determinants of health impact uptake of health information or disinformation?
- How to measure the link between the spread of online health misinformation and negative behavioral outcomes (from a health perspective)?
- How to use available data for infodemic surveillance and early alert?
- What measurement and evaluation tools can be developed for offline information flows? What would a holistic whole-of-communication evaluation system (online and offline) for infodemics management look like? How to evaluate different interventions?
- How can automated methods involving machine learning and artificial intelligence be used for pattern recognition and alerting?
- How do rumors spread across countries in a region?
- To what extent does misinformation relating to specific therapeutics result in adverse health impacts? [Feasibility: 12-18 months]
- How to assess which misinformation is most important to address (i.e. which misinformation is more likely to adversely impact individual health behavior)?
- What type of information contributes to an infodemic? Any differences between human-generated and machine-generated content?
- What are the possible sources to collect evidence in a timely manner?
- What are the most feasible applications of artificial intelligence (and machine/reinforcement learning techniques) to detect the origins and transmission vectors of mis/disinformation on selected social media platforms?
- What is the relationship between individual-level characteristics (e.g., socio-economic, trust in institutions, etc.), information consumption patterns and health-related behavior?
- What measurable factors should be used to determine the threshold for deciding if an intervention benefits and cost-effectiveness outweigh potential harms and unintended amplification?
- What post-event analysis methodology/framework can be applied to understand and track how the infodemic originated and spread?
- To what extent do people change behaviors based on misinformation?
- How does misinformation from different platforms, media, or channels foster behavior change?
- What are the linkages of misinformation with drivers for behavior and social change?
- What existing vulnerabilities (international, national, local) and narratives can be exploited by malicious actors in disinformation campaigns in the context of epidemics?
- How does media literacy mediate the adverse health impacts of misinformation? What are the policy implications?
- How can automated, anonymised methodologies (e.g. machine learning) help to overcome privacy risks associated with infodemic surveillance?
- What kind of change in online information-sharing can limit the spread of harmful health information? What is the most effective way to prevent the spread of harmful health information when regulating in different contexts?
- How can media literacy campaigns be designed to be more responsive to specific social settings?
- How should health systems prepare for prevention, monitoring, early warning and risk assessment of infodemic?
- To what extent are migrant populations (immigrants, refugees, asylum seekers, labour migrants, international students), in particular those irregular migrants within a country, affected by infodemics? To what extent does infodemics permeate within the ecosystems of how migrant communities access information on public health measures?
- How can health system assets be used to counter the impact of dissemination of misinformation/disinformation on different public health program? [Feasibility: ~12 months]
- How to harness community expertise and community organizations to develop contextually-relevant interventions?
- Which models, theories or methodologies can infodemic management research borrow from health literacy research?
- How can techniques such as 'nudge' messaging or random mixing of narratives from opposing camps help to overcome the escalation or entrenchment of harmful belief systems, as seen in existing social media 'filter bubbles'?
- How are infodemics associated with the mode of information accessing, understanding and spreading among different age group population?
- What are the best forms of public engagement for alerting citizens to the psychological influence of harmful online messaging and supporting critical evaluation?
- How to build a set of common requirements for public health agencies, healthcare providers and related institutions, to support a more effective response to misinformation during pandemics?
- Does access to universal health coverage act as lever for greater trust in public health institutions? Does it act as a means of infodemic resilience?
- How do pandemic conspiracy theories spread in different cultures and geo-political settings?
- What are the long-term effects of interventions against misinformation on the behavior of individuals and communities?
- How can health organizations and communicators (e.g., chief medical officers) strengthen their credibility?
- What policy interventions are relevant to infodemic management? Why?
- What are the effects of interventions to identify, prioritise, reduce the spread, and mitigate the misinformation consequences?
- What knowledge, skills and tools contribute to strengthen the resilience of individuals and ‘inoculate’ them against misinformation and disinformation? How can public health organisations, the media and the education sector contribute to strengthen that resilience?
- How is individual behavior affected by changing information content or transmission mode?
- What are use cases of diffusion of health information by gradients of quality and harmful impact, that can be used to research and build a knowledge base for infodemic management training of health and communication professionals?
- How can infodemic management be better coordinated between different levels of government, and what is the comparative advantage at each level?
- To what extent have member states included strategies on addressing/managing the impact of misinformation during a pandemic within their national pandemic preparedness and response plans?
- How to develop a real-time data collection tool to improve the effectiveness and timeliness of response to infodemics?
- Which actors should be considered as the "initiator" for building infodemiology capacity in a country (i.e. academia, Ministry of Health)? Why?
- What rapid alert/indications and warning system on infodemics should be in place? What organizations should take part in it?
- How to design interventions to be more responsive to specific geographical factors (including socio-cultural, political and historic)?
- How can the extent and the threat of infodemics be measured within and across borders, with which with reliable and validated instruments/tools?
- Which member states are comparable regarding their information structure and information behavior?
- How to harness innovative techniques such as crowdsourcing and AI pattern-detection methods to identify early warning signs of infodemics?
- How to further empower schools and higher education institutions in addressing critical thinking and knowledge of scientific thinking?
- What can be learnt from a comprehensive meta-analysis of research focused on aspects relevant to infodemic management?
- What is the estimated impact of misinformation on policy, with examples?
